# The outcome in patients with BRAF‐mutated metastatic melanoma treated with anti‐programmed death receptor‐1 monotherapy or targeted therapy in the real‐world setting

**DOI:** 10.1002/cam4.6982

**Published:** 2024-03-16

**Authors:** Jindřich Kopecký, Marek Pásek, Radek Lakomý, Bohuslav Melichar, Ivona Mrazová, Ondřej Kubeček, Monika Arenbergerová, Radmila Lemstrová, Alžběta Švancarová, Vojtěch Tretera, Alžběta Hlodáková, Kamila Žváčková

**Affiliations:** ^1^ Department of Clinical Radiotherapy and Oncology University Hospital in Hradec Kralove Hradec Kralove Czech Republic; ^2^ Department of Dermatovenereology, Third Faculty of Medicine Charles University and Kralovske Vinohrady University Hospital Prague Czech Republic; ^3^ Department of Comprehensive Cancer Care, Masaryk Memorial Cancer Institute and Faculty of Medicine Masaryk University Brno Czech Republic; ^4^ Department of Oncology, Faculty of Medicine and Dentistry Palacký University and University Hospital Olomouc Czech Republic; ^5^ Department of Oncology County Hospital České Budějovice Czech Republic

**Keywords:** BRAF mutation, immunotherapy, real‐world data, targeted therapy

## Abstract

**Background:**

Immunotherapy and targeted therapy are currently two alternative backbones in the therapy of BRAF‐mutated malignant melanoma. However, predictive biomarkers that would help with treatment selection are lacking.

**Methods:**

This retrospective study investigated outcomes of anti‐programmed death receptor‐1 monotherapy and targeted therapy in the first‐line setting in patients with metastatic BRAF‐mutated melanoma, focusing on clinical and laboratory parameters associated with treatment outcome.

**Results:**

Data from 174 patients were analysed. The median progression‐free survival (PFS) was 17.0 months (95% CI; 8–39) and 12.5 months (95% CI; 9–14.2) for immunotherapy and targeted therapy, respectively. The 3‐year PFS rate was 39% for immunotherapy and 25% for targeted therapy. The objective response rate was 72% and 51% for targeted therapy and immunotherapy. The median overall (OS) survival for immunotherapy has not been reached and was 23.6 months (95% CI; 16.1–38.2) for targeted therapy, with a 3‐year survival rate of 63% and 40%, respectively. In a univariate analysis, age < 70 years, a higher number of metastatic sites, elevated serum LDH and a neutrophil–lymphocyte ratio above the cut‐off value were associated with inferior PFS regardless of the therapy received, but only serum LDH level and the presence of lung metastases remained significant predictors of PFS in a multivariate analysis.

**Conclusions:**

Present real‐world data document the high effectiveness of immunotherapy and targeted therapy. Although targeted therapy had higher response rates, immunotherapy improved PFS and OS. While the prognostic value of LDH was confirmed, the potential use of blood cell count‐derived parameters to predict outcomes needs further investigation.

## 1 INTRODUCTION

Systemic therapy for patients with BRAF‐mutated melanoma is currently based on two different approaches, either targeted therapy (BRAF and MEK inhibition) or immunotherapy (anti‐programmed death receptor (PD)‐1 therapy alone or combined with anti‐cytotoxic T lymphocyte antigen (CTLA)‐4 therapy), both in the adjuvant (except for combined immunotherapy) or metastatic setting.

In light of recently published results from prospective trials and based on numerous retrospective studies with advanced melanoma, the perspective on the choice of targeted therapy and immunotherapy is becoming more apparent but still not well defined. Based on the results from a sequential prospective randomised phase II Secombit trial,^1^ a phase III DreamSeq trial^2^, ^3^ and RELATIVITY‐047,^4^ there is a tendency to favour immunotherapy combination in the first line. These results align with previous signals from other trials and subsequent analyses of subgroups with BRAF‐mutated patients.^5^, ^6^ Nevertheless, these clinical trials compare doublet immunotherapeutic agents to BRAF/MEK inhibitors. Because in most of these prospective and retrospective studies, the efficacy of the very same drugs in the second‐line therapy was worse, it is essential to maximise the clinical benefit of the first‐line therapy to delay the need for patient transition to the second‐line. However, given the lower response rate, it is uncertain whether first‐line anti‐PD1 monotherapy is superior to BRAF/MEK inhibition as there are patients who are unsuitable for combination ipilimumab–nivolumab or relatlimab–nivolumab. Another unmet need is the lack of predictive biomarkers that could help us guide the best treatment option in real‐world practice.

We performed a retrospective analysis of patients with BRAF‐mutated metastatic melanoma treated with BRAF/MEK inhibition therapy or anti‐PD‐1 monotherapy (nivolumab or pembrolizumab). Our study aimed to evaluate the efficacy, that is, progression‐free survival (PFS), overall survival (OS) and objective response rate (ORR) of anti‐PD‐1 monotherapy and BRAF/MEK inhibition therapy in patients with BRAF‐mutated metastatic melanoma. We also searched for potential prognostic parameters that could aid in choosing the optimal treatment modality in the real‐world setting.

## 2 MATERIALS AND METHODS

Consecutive treatment‐naive patients with BRAF‐mutated metastatic melanoma who started treatment between October 2012 and October 2021 in the University Hospital Hradec Kralove, University Hospital Olomouc, University Hospital Královské Vinohrady in Prague, County Hospital České Budějovice and Masaryk Memorial Cancer Institute were included in this retrospective study. All patients were indicated for therapy either with anti‐PD1 therapy (nivolumab or pembrolizumab) or BRAF/MEK inhibition therapy (dabrafenib/trametinib, vemurafenib/cobimetinib or encorafenib/binimetinib).

The principal inclusion criteria included (1) metastatic melanoma (excluding uveal melanoma) with measurable disease (according to the RECIST v1.1 criteria); (2) BRAF V600 mutation; (3) no previous systemic treatment for advanced disease (patients with prior adjuvant treatment were included if completed more than 6 months before the therapy for metastatic disease); and (4) indication for treatment with nivolumab, pembrolizumab, dabrafenib/trametinib, vemurafenib/cobimetinib and encorafenib/binimetinib in standard dose schedule. Patients were excluded from analysis if (1) treated in a clinical trial, (2) were without baseline clinical data or (3) had a missing evaluation of radiologic results.

The study was conducted according to the principles of the Declaration of Helsinki and with the approval of the local ethic committee.

### 2.1 Variables and radiologic assessments

We collected the laboratory data from the patient's medical record before the treatment initiation and 12 weeks after the start of therapy. The following parameters were of interest: white blood cell count (WBC), platelets count (PLT), absolute lymphocyte count (ALC), absolute neutrophil count (ANC) and absolute monocyte count (AMC). The patient characteristics were also collected (age, sex, adjuvant treatment, site of metastases, number of affected organs/sites, primary histology and serum lactate dehydrogenase (LDH) levels).

BCDR assessed included neutrophil–lymphocyte ratio (NLR), platelet–lymphocyte ratio (PLR), lymphocyte–monocyte ratio (LMR), systemic inflammation index (SII) and derived neutrophil to lymphocyte ratio (dNLR). NLR was calculated as NLR = ANC/ALC, PLR = PLT/ALC, LMR = ALC/AMC, SII = ANC × PLT/ALC and dNLR = ANC/(WBC‐ANC).

Tumour extent was assessed at baseline and then every 12 to 16 weeks. The response was classified according to the Response Evaluation Criteria in Solid Tumours v1.1 (RECIST). Objective response rate (ORR) was defined as complete response (CR) together with partial response (PR), and disease control rate (DCR) was defined as CR, PR together with stable disease (SD).

Progression‐free survival (PFS) was defined as an interval from the first treatment cycle to the first evidence of radiological/clinical disease progression or death from any cause. In patients treated with immunotherapy, the treatment continued after the first progression documented by imaging methods. A confirmation imaging method was performed within 4–8 weeks to rule out pseudoprogression. In case of confirmed disease progression, the date of the first imaging method demonstrating disease progression was used for the PFS calculation. PFS1 and PFS2 were used in the analysis of patients with sequential therapy. The definition of PFS1 was the same as for PFS. PFS2 was defined as the time from the initiation of the second‐line therapy to objective tumour progression on next‐line treatment or death from any cause. Overall survival (OS) was defined as an interval from the beginning of therapy to death (event) or the last follow‐up (censored). The cut‐off date for the analysis was 4 February 2022.

### 2.2 Statistical analysis

Descriptive statistics were used to summarise patient and treatment characteristics. The comparison of parameters between patients with anti‐PD1 or BRAF/MEK inhibitors was performed with the chi‐square, Fisher's exact and Mann–Whitney tests. The continuous data were dichotomised if feasible and meaningful.

Cox proportional hazards models for univariate and multivariate analyses were used to investigate associations of selected clinical variables with survival (PFS and ORR) and adjusted for baseline characteristics (therapy, gender, age, presence of synchronous metastatic disease, LDH and individual blood cell count‐derived ratios). All parameters with a *p*‐value <0.1 following univariate analysis were entered into the multivariate model. Results were presented as hazard ratios (HR) with 95% confidence intervals (CIs). The Kaplan–Meier method with Rothman's 95% confidence intervals (CI) was used to estimate OS and PFS survival curves.

A survival receiver operating characteristic (ROC) analysis was adopted to identify potential cut‐offs that optimally stratify patients into risk groups. Optimal thresholds for blood cell count‐derived ratios were determined based on literature reviews^7^, ^8^, ^9^, ^10^, ^11^, ^12^, ^13^ and our previous work, where we used those cut‐offs and verified their applicability.^14^ The cut‐off values were set as follows: NLR 3, PLR 160, LMR 2, SII 800 and dNLR 1,9, respectively. Differences were considered statistically significant when the *p*‐value was <0.05. The statistical analysis was performed by MedCalc® Statistical Software version 20.110 (MedCalc Software Ltd, Ostend, Belgium; https://www.medcalc.org; 2022).

## 3 RESULTS

### 3.1 Patient characteristics

**FIGURE 1:**
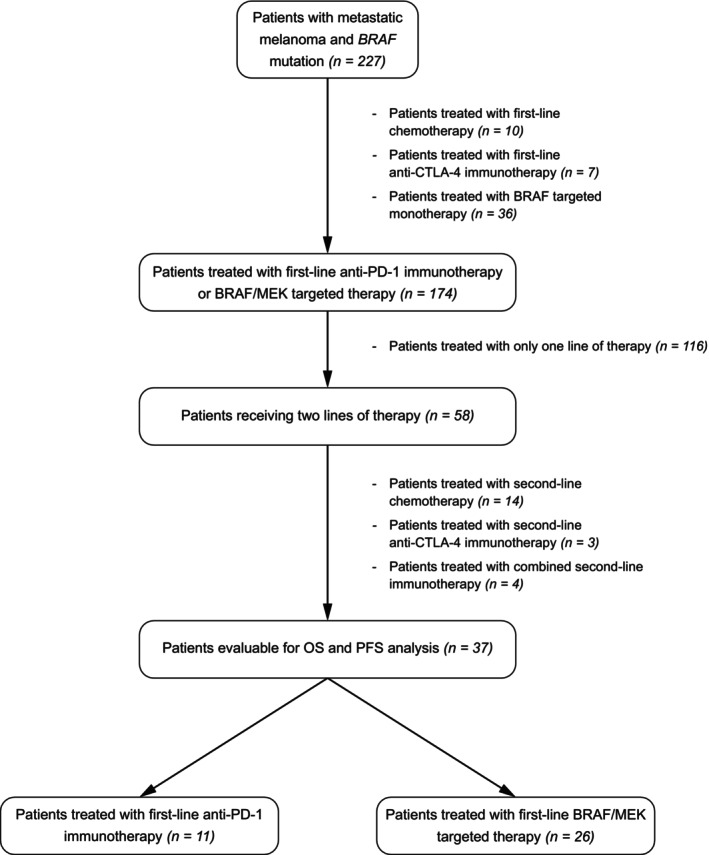
Patient enrolment.

Among 227 patients harbouring BRAF mutation in the database and treated with targeted therapy or immunotherapy (Figure 1), 174 who fulfilled inclusion criteria were included in the OS, PFS, ORR analysis and univariate and multivariate analyses. Sixty‐five patients were treated with anti‐PD1 (7 patients received pembrolizumab and 58 nivolumab), and 109 patients with BRAF V600E/K were treated with BRAF/MEK inhibitors (9 patients received vemurafenib/cobimetinib, 13 encorafenib/binimetinib and 87 dabrafenib/trametinib). The median age of all patients was 66 years (range 24–91), and the performance status at the time of therapy was ECOG 0 or 1. Twenty‐four patients were pretreated with adjuvant therapy (7 patients before immunotherapy and 17 before BRAF/MEK inhibition). Thirteen patients had interferon alfa2b (4 in nivolumab and 9 in targeted therapy group), six nivolumab (all before BRAF/MEK inhibition), two patients had combination dabrafenib/trametinib (all before anti‐PD‐1 monotherapy), one patient had vaccine therapy before BRAF/MEK inhibition, and the patient with adjuvant chemotherapy was treated with anti‐PD‐1 monotherapy after progression. One patient had adjuvant radiotherapy before palliative therapy with BRAF/MEK inhibition.

Fifty‐eight patients received subsequent systemic therapy, including 37 patients with a sequence of anti‐PD‐1 therapy and combined BRAF MEK inhibitors or vice versa (Figure 1). When comparing baseline characteristics (Table 1), there was a significant difference in the number of involved metastatic sites, with a higher rate in patients treated with targeted therapy.

**TABLE 1:** Baseline clinical and demographic characteristics.

	Immunotherapy		Targeted therapy		*p*‐value
	*n* = 65	%	*n* = 109	%	
Gender					0.52
Female	26	40	38	35	
Male	39	60	71	65	
Age					0.87
≥70 years	27	42	43	39	
<70 years	38	58	66	61	
Median (years, range)	65 (30–91)		66 (24–86)		0.91
BRAF mutation					0.0005^*^
V600E	39	60	58	53	
V600K	17	26	13	12	
Other (V600A, V600R G469K etc.)	2	3	0	0	
The type of mutation not assessed	7	11	38	35	
Synchronous metastatic disease (cut‐off 3 months)					0.61
Yes	21	32	30	28	
No	44	68	79	72	
Stage M (AJCC 8th version)					0.015^*^
M1a	26	40	26	24	
M1b	13	20	12	11	
M1c	20	31	55	50	
M1d	6	9	16	15	
Number of involved organs					0.0004^*^
1	26	40	18	17	
2 and 3	32	49	58	53	
≥4	7	11	33	30	
Metastatic site					0.75
Lung	24	19	52	18	
Liver	9	7	32	11	
Lymph nodes	34	26	75	26	
Subcutaneous and soft tissue	31	24	55	19	
Brain	6	5	16	5	
Bone	11	9	22	8	
Other	14	11	40	14	
Adjuvant therapy					0.49
Yes	7	11	17	16	
No	58	89	92	84	
LDH ≥ ULL					0.11
Yes	25	38	56	51	
No	39	60	52	48	
Not assessed	1	2	1	1	
LDH (median, range)	3.37	1.1–20.5	3.84	1.9–23.2	0.11
Time to metastatic disease [median, months] (range)	25.8 (14.9–35)		17 (11–24)		0.85

Abbreviations: LDH, lactate dehydrogenase; ULL, upper limit level; *statistically significant.

We did not observe any new toxicity signals for immunotherapy and targeted therapy. Twenty‐two patients stopped therapy due to toxicity; 11 are still alive (7 in the immunotherapy arm and 6 in the BRAF/MEK inhibition arm).

Eleven patients with anti‐PD‐1 monotherapy had to stop therapy due to toxicity grade 3 and higher (4× pneumonitis, 2× hepatitis, renal toxicity, colitis and diabetes mellitus, neurotoxicity and myositis). Before stopping immunotherapy, three reached CR, five PR, and one patient SD and PD. Five patients needed subsequent therapy (2× chemotherapy, 1× nivolumab, 2× dabrafenib/trametinib).

Eleven patients treated with BRAF/MEK inhibition in the first line had to stop therapy because of toxicity (febrile 5×, 2× phototoxicity, ocular toxicity, 2× renal toxicity, rhabdomyolysis and cardiotoxicity). Before stopping for toxicity, two patients reached CR, four PR, two SD and one PD. Two stopped before the first radiologic evaluation. Five patients received the subsequent therapy (3× nivolumab, ipilimumab, nivolumab/ipilimumab).

### 3.2 Progression‐free survival and objective response rate

At the cut‐off date, the median follow‐up was 34 months with a minimum of 4 months. One hundred sixteen patients have progressed (77 with targeted therapy and 39 with immunotherapy). Only 58 patients received second‐line therapy before the cut‐off date. At the time of analysis, 39 patients were still on the first‐line therapy (24 patients treated with targeted therapy and 15 treated with immunotherapy).

**FIGURE 2:**
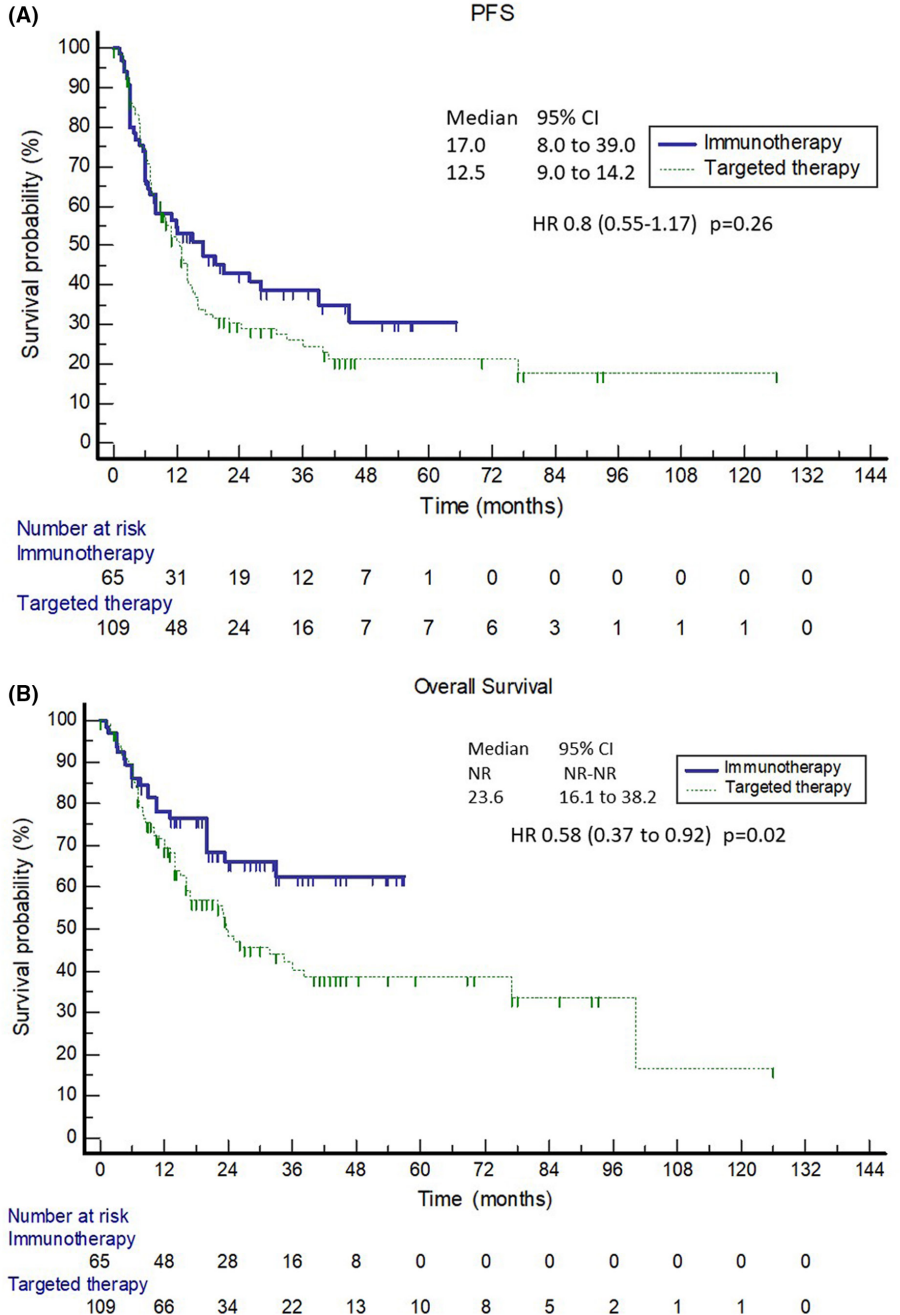
(A) Progression‐free survival (PFS) and (B) overall survival for patients with immunotherapy and targeted therapy within the first line, CI, confidential interval; HR, hazard ratio.

The median PFS was 17.0 months (95% CI; 8–39) and 12.5 months (95% CI; 9–14.2) in patients treated with first‐line immunotherapy and targeted therapy, respectively (Figure 2A). There was no statistically significant difference between both groups with HR 0.8 (95% CI; 0.55–1.17; *p* = 0.26). The 1‐, 2‐ and 3‐year PFS rate was 53%, 43% and 39% for anti‐PD‐1 therapy and 51%, 30% and 25% for targeted therapy.

We observed a higher ORR in patients treated with targeted therapy compared to immunotherapy, 72% vs 51%, with a similar proportion of CR in both groups (Table 2). The DCR also favoured targeted therapy. Conversely, the percentage of patients who progressed after achieving disease control was significantly higher in patients treated with targeted therapy than with immunotherapy (64% vs. 45%) (Table S1).

**TABLE 2:** Best tumour responses.

	Immunotherapy		Targeted therapy		*p*‐value
	*n* = 65	%	*n* = 109	%	0.008
CR	17	26	24	22	
PR	16	25	54	50	
SD	14	22	12	11	
PD	18	27	19	17	
ORR	33	51	78	72	
DCR	47	73	90	83	

Abbreviations: CR, complete response; DCR, disease control rate; ORR, objective response rate; PD, progressive disease; PR, partial response; SD, stable disease.

**FIGURE 3:**
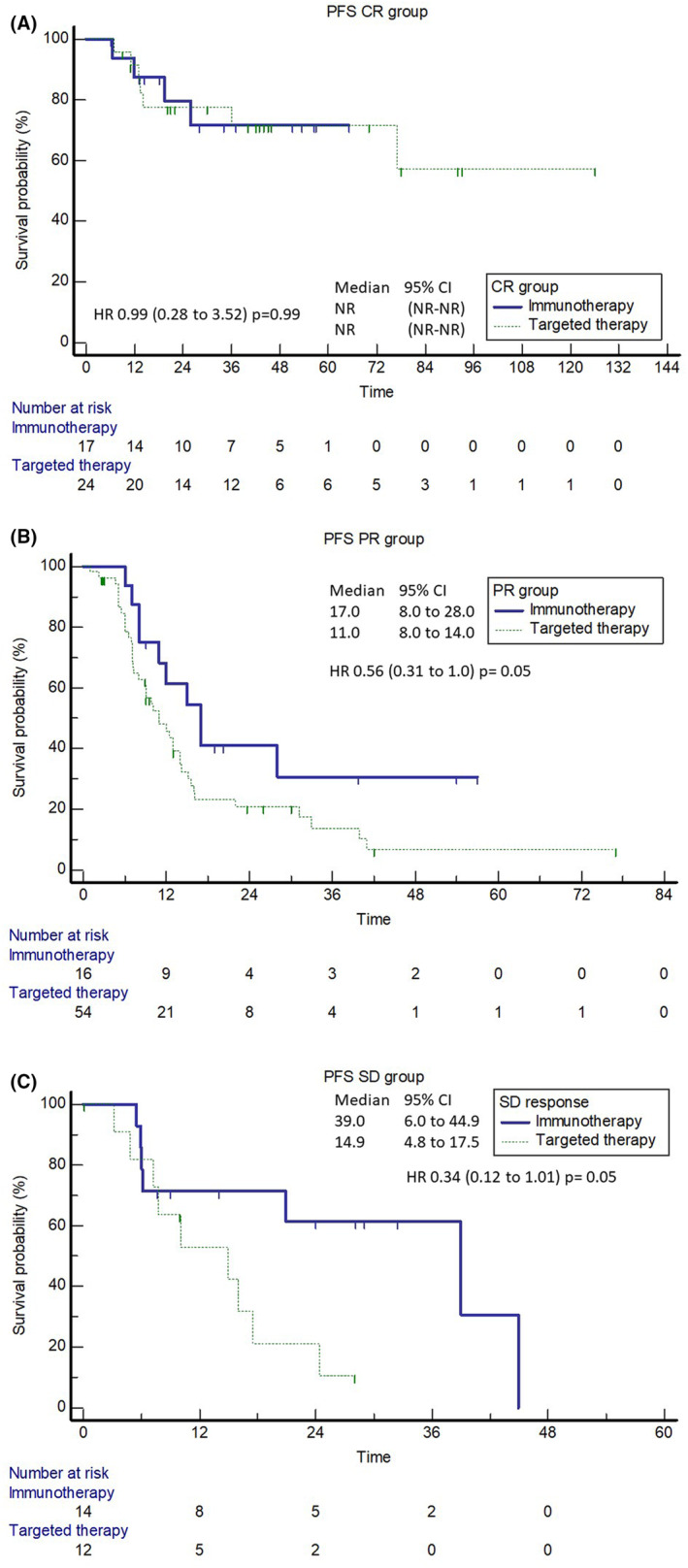
Duration of response in patients (A) with complete response (CR); (B) with partial response (PR); and (C) with stable disease (SD).

The duration of disease control in patients with PR and SD was superior in patients treated with immunotherapy, HR 0.56 (95% CI; 0.31–1.0; *p* = 0.05) for PR and HR 0.34 (95% CI; 0.12–1.0; *p* = 0.05) for patients with SD. Patients with CR had a similar median duration of response as for immunotherapy as targeted therapy (Figure 3A–C).

### 3.3 Overall survival

At the time of analysis, 78 patients have died. We observed a statistically significant difference with prolonged OS in the immunotherapy group (Figure 2B). The median OS in patients treated with immunotherapy has not been reached, while the median OS in patients treated with targeted therapy was 23.6 months (95% CI; 16.1–38.2), with a 3‐year survival of 63% and 40% for immunotherapy and targeted therapy, respectively.

### 3.4 Blood cell count‐derived ratios (BCDRs)

We analysed all BDCRs for 64 patients (one patient was excluded for missing differential blood count) with anti‐PD‐1 therapy and 109 patients with targeted therapy. In the group of patients treated with targeted therapy, we observed a higher frequency of unfavourable profiles of all BDCRs except dNLR and PLR (Table 3).

**TABLE 3:** Baseline characteristics of blood cell count‐derived ratios.

	Immunotherapy		Targeted therapy double		*p*‐value
	*n* = 64	%	*n* = 109	%	
NLR					0.01^*^
≥ cut‐off	22	34	59	54	
< cut‐off	42	66	50	46	
NLR (median, range)	2.7	0.6–11.2	3.1	0.5–18.0	0.09
SII					0.04^*^
≥ cut‐off	23	36	57	52	
< cut‐off	41	64	52	48	
SII (median, range)	669.4	12.6–2479.3	848.9	55.0–7301.2	0.01^*^
dNLR					0.44
≥ cut‐off	29	45	56	51	
< cut‐off	35	55	53	49	
dNLR (median, range)	1.8	0.2–5.5	1.9	0.4–12.5	0.17
LMR					0.01^*^
≥ cut‐off	55	86	75	69	
< cut‐off	9	14	34	31	
LMR (median, range)	2.9	0.9–7.0	2.5	0.8–11.7	0.02^*^
PLR					0.07
≥ cut‐off	21	33	51	47	
< cut‐off	43	67	58	53	
PLR (median, range)	128.4	1.9–274.9	155.5	35.2–668.6	0.01^*^

Abbreviations: dNLR, derived neutrophil–lymphocyte ratio; LMR, lymphocyte–monocyte ratio; NLR, neutrophil–lymphocyte ratio; PLR, platelet–lymphocyte ratio; SII, systemic inflammation index; *statistically significant.

To assess the impact of each BCDR on PFS, we performed a Kaplan–Meier analysis for the immunotherapy and targeted therapy groups separately. All BDCRs defined as unfavourable (except for LMR and PLR) were associated with significantly inferior PFS (Figure S1A–F) in the immunotherapy group. Conversely, in the targeted therapy group, only serum LDH level was significantly associated with adverse PFS (Figure S2A–F).

We observed no statistically significant differences in the Kaplan–Meier analysis for PFS comparing BCDRs between targeted therapy and immunotherapy (Table S2). Nevertheless, we observed a trend of prolonged median PFS in patients treated with immunotherapy if the baseline BDCR were below the cut‐off. The most pronounced difference was for dNLR (44.9 vs. 14 months) and SII (25.8 vs. 14 months).

### 3.5 Comparing CR/PR and PD responses

We compared two subgroups of patients, that is, those showing response to therapy (i.e., CR or PR) and patients progressing on therapy. We compared the basic clinical characteristics and laboratory parameters. No statistically significant differences were identified between these two subgroups (Table S3).

Next, we compared the targeted therapy and immunotherapy group objective response (CR, PR, PD). Patients who achieved CR and were treated with immunotherapy had, in comparison with patients with BRAF/MEK inhibition therapy, higher incidence of nodular (65% vs. 29%) and superficial spreading histology (24% vs. 8%); the diagnosis was performed more often from primary lesions (12% vs. 54%), and they had higher proportion of BRAF V600K mutations (35% vs. 4%). In the group of patients with PR response, the only statistical difference was seen for LDH elevation with lower frequency in the immunotherapy group (31% vs. 61%).

For blood cell count‐derived parameters comparing groups of patients with immunotherapy and BRAF/MEK therapy, we observed differences in the frequency of NLR and SII above cut‐off levels (12% vs. 46% and 18% vs. 50%, respectively) in patients with CR. There was a difference in LMR frequencies (94% vs. 63%) in patients with PR. In the case of PD, the only statistically significant differences were observed in a higher proportion of lymph node involvement (14% vs. 27%) and a higher rate of patients with dNLR above the cut‐off value (29% vs. 63%). All results are presented in Table S4.

### 3.6 Univariate and multivariate analyses

In the univariate analysis, age < 70 years, higher number of metastatic sites, elevated serum LDH and NLR above the cut‐off value were associated with shorter PFS. Further, lung and liver metastases were significant predictors of shorter PFS. However, only serum LDH level and the presence of lung metastases remained significant predictors of PFS in the multivariate analysis (Table 4).

**TABLE 4:** Univariate and multivariate analysis for progression‐free survival.

	Univariate analysis			Multivariate analysis		
	HR	95%CI	*p*‐value	HR	95%CI	*p*‐value
Type of therapy (IO vs. BRAF)	0.8	0.54–1.18	0.26	—	—	—
Gender (women vs. men)	1.09	0.75–1.59	0.65	—	—	—
Age ≥ 70 (yes vs. no)	0.65	0.43–0.98	0.04^*^	0.72	0.47–1.09	0.12
Initial metastatic (yes vs. no)	1.12	0.75–1.67	0.59	—	—	—
Number of involved sites ≥4 (yes vs. no)	1.73	1.15–2.6	0.008^*^	0.95	0.56–1.60	0.84
Adjuvant therapy (yes vs. no)	1.37	0.83–2.27	0.22	—	—	—
LDH ≥ ULL (yes vs. no)	1.87	1.30–2.71	0.0008^*^	1.63	1.10–2.41	0.01^*^
NLR1 ≥ 3 (yes vs. no)	1.47	1.02–2.13	0.038^*^	1.19	0.81–1.74	0.38
LMR1 ≥ 2 (yes vs. no)	0.81	0.53–1.22	0.32	—	—	—
PLR1 ≥ 160 (yes vs. no)	1.22	0.84–1.76	0.29	—	—	—
SII1 ≥ 800 (yes vs. no)	1.39	0.97–2.01	0.07	—	—	—
dNLR1 ≥ 1,9 (yes vs. no)	1.43	0.99–2.06	0.058	—	—	—
Lung metastases (yes vs. no)	1.75	1.21–2.53	0.0028^*^	1.71	1.12–2.62	0.01^*^
Liver metastases (yes vs. no)	1.68	1.12–2.54	0.013^*^	1.33	0.82–2.17	0.25
Lymph node metastases (yes vs. no)	1.04	0.72–1.51	0.83	—	—	—
Subcutaneous metastases (yes vs. no)	1.36	0.94–1.96	0.09	1.65	1.09–2.50	0.01^*^
Brain metastases (yes vs. no)	1.33	0.77–2.28	0.31	—	—	—
Bone metastases (yes vs. no)	1.32	0.84–2.07	0.23	—	—	—

Abbreviations: BRAF, BRAF inhibition; CI, confidential interval; dNLR, derived neutrophil–lymphocyte ratio; IO, immunotherapy; LMR, lymphocyte–monocyte ratio; NLR, neutrophil–lymphocyte ratio; PLR, platelet–lymphocyte ratio; SII, systemic inflammation index; ULL, upper limit level; *statistically significant.

We also performed univariate and multivariate analyses for patients treated with targeted therapy and immunotherapy separately to evaluate the impact of each parameter on PFS within these subgroups. In the targeted therapy subgroup, age was no longer associated with shorter PFS in the univariate analysis. On the other hand, the presence of brain metastases showed a significant impact on PFS in the univariate analysis. However, in the multivariate analysis, only serum LDH and lung and brain metastases were associated with shorter PFS (Table S5).

In the immunotherapy subgroup, the result of univariate analysis identified age, serum LDH and NLR as predictors for PFS, similar to the general study population. Furthermore, dNLR and subcutaneous and lymph node metastases were identified as additional negative PFS predictors in this subgroup. In contrast to the general population, liver and lung metastases did not impact PFS in the immunotherapy subgroup. In the multivariate analysis, only age, serum LDH level and the presence of subcutaneous metastases had a statistically significant impact on PFS and demonstrated in shorter time intervals (Table S6).

### 3.7 Sequential therapy

The second‐line therapy was indicated in 58 patients. The therapy consisted of chemotherapy, anti‐PD‐1 or anti‐CTLA‐4 monotherapy, combined immunotherapy and BRAF/MEK inhibition therapy (see Figure 1). The rest of the patients were not candidates for any further therapy due to rapid progression or deterioration of clinical status, and palliative care was indicated. The 37 patients receiving either anti‐PD‐1 monotherapy or BRAF/MEK inhibition in the second line were included in further analysis. More than two‐thirds were treated with targeted therapy in the first line (25 with dabrafenib/trametinib and one with encorafenib/binimetinib). Eleven patients were treated with immunotherapy in the first line. These groups had no statistically significant differences in the clinical and demographic parameters. There was a slightly higher proportion of male patients and patients with more affected organs/sites in the group with targeted therapy in the first line (Table 5). To the censoring date, the median follow‐up was 77 months, ranging between 6 and 77 months.

**TABLE 5:** Baseline characteristics for patients with sequential therapy.

	Sequence IO‐TT		Sequence TT‐IO		*p*‐value
	*n* = 11	%	*n* = 26	%	
Gender					0.08
Female	7	64	8	31	
Male	4	36	18	69	
Braf mutation					0.16
V600E	9	82	16	62	
V600K	2	18	3	12	
Not assessed	0	0	7	26	
Synchronous metastatic disease					0.12
Yes	5	45	5	19	
No	6	55	21	81	
Number of involved organs					0.07
1	3	27	4	15	
2–3	8	73	13	50	
≥4	0	0	9	35	
Metastatic site					0.24
Lung	2		12		
Liver	0		9		
Lymph nodes	6		19		
Subcutaneous	9		14		
Brain	0		2		
Bone	1		4		
Other	3		15		
Adjuvant therapy					0.65
Yes	1	9	6	23	
No	10	91	20	77	
LDH ≥ ULL					0.49
Yes	4	36	13	50	
No	7	64	13	50	
Age [median, years] (range)	67 (38–76)		64 (38–82)		0.85
Time to metastatic disease [median, months] (range)	11 (0–132)		15 (0–171)		0.35

Abbreviations: IO, immunotherapy; LDH, lactate dehydrogenase; TT, targeted therapy; ULL, upper limit level.

The median PFS1 for immunotherapy and targeted therapy was 6.0 months and 8.8 months, respectively. One‐year PFS was 39% and 27% for immunotherapy and targeted therapy. The difference in median PFS was not statistically significant, with an HR of 1.21 (95% CI; 0.57–2.60).

At the time of analysis, medians for PFS2 have not been reached. However, there was a significant improvement with an HR of 0.30 (95% CI; 0.09–0.99) for the sequence immunotherapy‐targeted therapy. One‐year PFS2 was 100% and 53% for the sequence immunotherapy‐targeted therapy and targeted therapy‐immunotherapy, respectively.

The median OS for the sequence of immunotherapy‐targeted therapy has not been reached, and the median OS for the patients treated with targeted therapy in the first line, followed by immunotherapy, was 38.2 months (95% CI; 17.0–77.0). The difference was not statistically significant due to a small sample size (HR 0.34; 95% CI; 0.1–1.28; *p* = 0.11). However, 1‐, 2‐ and 3‐year survivals were 100%, 100% and 66% when immunotherapy was used in the first line and 85%, 57% and 57% for the group with targeted therapy in the first line.

## 4 DISCUSSION

The therapeutic landscape and optimal treatment sequence for advanced and/or metastatic melanoma was less clear at the time point when patients from our study population underwent therapy. However, new available data shed more light on the optimal treatment sequence. Based on clinical trial results, we currently have two fundamental treatment options, targeted therapy^15^, ^16^, ^17^ and immunotherapy.^18^, ^19^, ^20^ These new therapeutic approaches have significantly improved the outcome of metastatic melanoma patients. Although there are signals favouring doublet immunotherapy over BRAF/MEK inhibition therapy in the first‐line setting,^1^, ^2^ choosing between anti‐PD‐1 monotherapy and targeted therapy for treatment‐naive patients with BRAF‐mutant disease is still uncertain because we lack the data from head‐to‐head trials. Therefore, the data from the real‐world setting are needed to inform the treatment selection.

We performed a retrospective study on 174 patients with BRAF‐mutated melanoma. To the best of our knowledge, this is one of the largest cohorts of patients focusing on this specific group of patients treated outside of a clinical trial. It is essential to explore the outcomes of patients in the real world because clinical trials enrol patients with specific disease characteristics based on certain pre‐defined eligibility criteria, often not met in daily clinical practice.^21^ A recently published paper retrospectively analysed nearly two thousand patients with metastatic BRAF‐mutated melanoma. However, the paper focused more on the impact of the therapy sequence, concluding that the efficacy of second‐line therapy is poor compared with first‐line therapy.^22^ Nevertheless, there are countries where the therapeutic options are still limited without the possibility of combinational therapeutic regimes or the chance to use targeted therapy or immunotherapy in the second line. From this perspective, the present work has clinical relevance in the search for optimal first‐line therapy.

Present data on ORR and PFS are consistent with the previously reported targeted therapy clinical trial results. In the three principal clinical trials of BRAF and MEK inhibitors, ORR ranged between 64% and 71%, median PFS and 3‐year PFS rates were 12–14 months and 22%–28%, respectively.^23^, ^24^, ^25^, ^26^ On the other hand, we observed marked differences for the anti‐PD‐1 therapy, with median PFS being more than twice longer than reported for nivolumab in melanoma patients with either non‐mutated tumours (5.1 months) in the Checkmate 066 trial^27^ or irrespectively of BRAF tumour status (5.6 months for BRAF mutated and 6.9 months in unselected patients) in the Checkmate 067 trial.^5^ The present PFS results were even better than for pembrolizumab in patients without prior therapy, as reported in the Keynote 006 trial (11.6 months).^6^ The ORR and 3‐year PFS observed in the present retrospective study were also slightly superior to the clinical trials.

A selection bias might partially explain superior outcomes observed in the patients treated with immunotherapy. Based on baseline characteristics, patients treated with anti‐PD‐1 monoclonal antibodies in the present cohort had lower tumour burden (expressed as initial serum LDH level and the number of involved organs), and the present study population consisted of exclusively BRAF‐mutated patients. However, there were no marked differences in the baseline characteristics between the clinical trial populations (named above) and the present cohort. The presence of brain metastases was slightly higher in the present cohort (5%) compared to the clinical trials (2%–3%, except for the Keynote 006 trial, where 8%–13% of patients had brain metastases).^6^, ^19^, ^28^, ^29^ Similarly to the clinical trials with BRAF/MEK inhibitors,^15^, ^16^, ^17^ we observed a high activity of targeted therapy even in patients with unfavourable prognostic characteristics (high serum LDH levels, presence of brain metastases). Another possible explanation for better results of immunotherapy in comparison with targeted therapy presented in real‐world data studies, in general, might be due to different evaluations of disease progression, where in the case of confirmed pseudoprogression, the patient is counted as a non‐progressor, which is different to randomised clinical trials where the classical RECIST 1.1. were applied.

The OS results in patients treated with first‐line targeted therapy in the present cohort were consistent with those reported in the clinical trials, where the median OS was 22–33 months and the 3‐year OS was 38%–47%.^23^, ^24^, ^25^, ^26^ However, the median OS has not been reached in patients treated with immunotherapy, and the 3‐year OS was slightly higher than presented in the previous clinical trial population with anti‐PD‐1 monotherapy (50% vs. 60%).^6^, ^19^, ^28^ These data and data from the therapy indicate a clear trend favouring immunotherapy in the first‐line setting, with an increase of ~20% in the 3‐year OS rate. This is consistent with the results of the Dreamseq trial, where this difference was a reason for the premature stop of patient enrolment,^2^ although patients in the present retrospective study were treated with anti‐PD‐1 monotherapy.

A disbalance in the disease burden between the immunotherapy and targeted therapy groups might explain this difference in the present retrospective study. There are clear signals that patients with low‐burden disease have higher chances of treatment efficacy regardless of whether it is immunotherapy^30^, ^31^, ^32^ or targeted therapy.^33^, ^34^ Nevertheless, a possible explanation for better immunotherapy outcomes might be the duration of tumour response. In the present study, we observed that only patients achieving CR had a probability of long‐term response in the targeted therapy group. Conversely, even patients with PR and SD achieved long‐term disease control in the immunotherapy group. This phenomenon was also demonstrated in clinical trials with dabrafenib/trametinib, as patients with CR showed 5‐year OS rates of 71%, while patients with SD had a 5‐year OS rate of only 16%.^24^ Similar results were observed with vemurafenib/cobimetinib therapy.^35^

On the other hand, long‐term responses were also observed in patients achieving PR and SD who were treated with immunotherapy.^5^ As a next step, we decided to compare patients achieving response to therapy (CR or PR) to those showing PD regarding baseline clinical and laboratory characteristics. However, no significant differences were observed. Only a statistical trend towards difference in serum LDH level and metastatic site involvement was observed when comparing patients with CR and PD.

The most burning issue in the therapy of metastatic BRAF‐mutated melanoma is the scarcity of biomarkers predictive of therapy outcome. There are three principal directions in which biomarkers might be investigated: tumour tissue‐derived biomarkers (mutation load, microsatellite instability, gene signatures), tumour microenvironment‐based biomarkers (PD‐L1, LAG‐3 expression or presence of tumour infiltrated lymphocytes) and biomarkers associated with the host (microbiome and peripheral blood biomarkers).

Given the retrospective design of our study and the fact that knowledge of PD‐L1 expression is not necessary to administer targeted therapy or immunotherapy, we could not evaluate its impact on treatment results or identify if it could help us choose the best therapy. Until today, there are conflicting results regarding PD‐L1 expression and therapeutic outcomes. The reason for such conflicting results might be due to the use of various PD‐L1 immunohistochemical assays, different evaluations of PD‐L1 expression. Not to forget that PD‐L1 is an inducible parameter, and its expression might differ in primary and metastatic lesions.^36^, ^37^ Although there seems to be a correlation between PD‐L1 expression and the likelihood of therapeutic outcome, therapeutic activity has been observed even in patients with low PD‐L1 expression.^5^ Nevertheless, the knowledge of PD‐L1 expression might be helpful if we have to choose between monotherapy and combined immunotherapy when the patients with negative PD‐L1 expression may obtain a more significant benefit from the combination immunotherapy than from anti‐PD‐1 monotherapies.^5^

The parameters from peripheral blood are the easiest to obtain and seem to be good candidates as biomarkers to determine the possible outcome and monitor treatment effectiveness. However, interpreting such results might be challenging, limiting their use in clinical practice. Some signals show differences in the baseline of some BCDRs between patients with localised and metastatic disease.^38^

In recent years, several studies have tried to identify the impact of different BCDRs on the therapeutic outcome.^7^, ^8^, ^39^ We chose parameters that are easily obtainable in daily routine and do not need additional procedures. Some BCDRs have recently been shown to be highly sensitive biomarkers. Their usefulness has been proven as a prognostic factor in several cancers. However, there are only a few studies on melanoma patients treated with immunotherapy.^40^, ^41^, ^42^, ^43^, ^44^, ^45^ Nevertheless, most of these studies evaluated NLR, with limited investigations of other ratios, including PLR, LMR, SII and dNLR.

BCDR indirectly reflect the state of inflammation. Inflammation is crucial in the pathophysiology of many disorders, including cancer.^46^ These parameters indirectly mirror the inflammatory process. It is supposed that these changes in peripheral blood may reflect the presence of lymphoid cells in the tumour environment, which may influence the therapeutic results. A neutrophilic response in cancer, which might be mirrored as high NLR from peripheral blood, is associated with a poor prognosis. Indeed, it can inhibit the immune system by suppressing the cytotoxic activity of T cells.^47^ Similarly, high PLR correlates with a worse prognosis because of the ability of platelets to participate in the inflammatory reaction by releasing growth factors and increasing angiogenesis.^48^ Monocytes which serve as a source of dendritic cells and tumour‐associated macrophages are considered to promote tumorigenesis, thereby driving the negative impact on melanoma mirrored as low LMR in peripheral blood.^49^

We observed that patients with unfavourable BCDR showed similar median PFS when treated with immunotherapy or targeted therapy. On the other hand, in the case of favourable baseline BCDR values, the median PFS was almost twice as long in patients treated with immunotherapy compared to those treated with targeted therapy, although not statistically significant (25 months in the immunotherapy group and 14 months in the targeted therapy group, only for dNLR the median PFS was 44 months for immunotherapy). However, the only consistent parameter associated with PFS in a multivariate analysis was serum LDH level. When we looked for a correlation between LDH and BCDRs, we found positive correlations between LDH and NLR, PLR, SII (ρ between 0.29–0.30, *p* = 0.0001) and negative correlation with LMR (ρ = 0.24, *p* = 0.001). However, these results do not confirm any causality. Contrarily, this was expected since LDH and BCDRs are linked to disease burden. Although BCDRs have the potential to be good prognostic markers, their disadvantage is that there are no stable cut‐off levels, as in the case of LDH.

Consistently to our previous study, patients with favourable BCDR treated with immunotherapy had better PFS than other subgroups.^14^ In contrast to previous results, patients with unfavourable BCDRs treated with BRAF inhibitors had better results than the immunotherapy group. We believe this might be explained by different populations and fewer patients in the previous study.^14^ Disproportions in baseline BCDRs between the subgroups might provide another explanation, although there was no significant difference in the proportion of patients with baseline LDH over ULL.

Considering all data from clinical trials and the present results, we can postulate that anti‐PD‐1 monotherapy and BRAF/MEK inhibition therapy in the first‐line setting are effective. However, there remains an important question, which treatment sequence is superior to the other. During the last 2 years, at least two randomised clinical trials have addressed this question.^2^, ^50^ Both of these trials favoured the sequence of immunotherapy followed by targeted therapy. In light of these results combined with other real‐world studies,^34^ it should be emphasised that the proper therapy sequence is crucial in terms of OS. Even in small numbers of patients and the use of anti‐PD‐1 monotherapy, we saw the discrepancy between PFS and PFS2 favouring sequence of immunotherapy followed with targeted therapy despite all biases as different tumour burden or pretreatment with adjuvant therapy. Several factors, such as an early switch to targeted therapy in case of primary resistance to immunotherapy, might cause this.

On the other hand, if targeted therapy fails, we might anticipate the immunosuppressive tumour environment to prevail. It has been demonstrated that BRAF inhibitors can potentiate immune activity.^51^, ^52^, ^53^ However, this effect is time‐dependent, and with a longer duration of therapy, this phenomenon might change to an immune suppressive phenotype.^52^, ^54^, ^55^ This is well demonstrated and translated in clinical trials where the tumour response of immunotherapy is much lower in the second line after targeted therapy; meanwhile, the response to targeted therapy is more or less the same in the second and the first‐line setting.^2^, ^50^ Our data are in agreement with previous findings from subanalysis in clinical trials KEYNOTE 001^56^ and 006.^57^ Treatment with pembrolizumab showed a reduced ORR in patients previously treated with targeted therapy^56^ and, conversely, longer PFS if not pretreated with targeted therapy.^57^

The present retrospective study has several limitations. First, given the study design, some patients were excluded from the analysis, such as patients treated with combination immunotherapy, resulting in possible selection bias. In the Czech Republic, the reimbursement of ipilimumab and nivolumab combination therapy is restricted to the patients fulfilling at least one of the following criteria: (1) patient's LDH level is higher than ULN; (2) patient has disease stage M1b or higher; (3) patient has two or more organs affected by metastases; and (4) patient has mucosal melanoma. These factors are considered to be unfavourable in general. So, excluding those patients could be a reason for excellent outcomes for immunotherapy, especially if we could find those unfavourable conditions more frequent in the targeted therapy group. A rather heterogeneous cohort of patients has been treated with different treatment regimens with differences in activity. Multiple analyses were performed in subgroups of limited size and statistical power.

We believe that all data from randomised clinical trials and the real‐world evidence including the present data should be considered when discussing treatment strategies in patients harbouring BRAF mutation to select a tailored therapy. In the real‐world setting, we should remember what we want to achieve with the intervention. Based on available data, it seems that targeted therapy should be the choice if a major tumour response and, in particular, symptom control is the goal. Conversely, immunotherapy seems to be the best choice if we want a durable and solid tumour response. It should also be remembered that a response to immunotherapy after the failure of targeted therapy is worse than a response to targeted therapy after immunotherapy.^2^, ^58^, ^59^ Combining these two advantages (rapid and durable response) was the reason for performing clinical trials with triplet therapy, combining immunotherapy with targeted therapy. There was a hope that with this approach, we could combine the high therapeutic response of targeted therapy with the long‐lasting effectiveness of immunotherapy. However, out of the three published trials, only one showed statistically improved PFS.

Nevertheless, in an indirect comparison, HR and a 2‐year PFS seemed similar within the three studies.^60^, ^61^, ^62^ These trials demonstrated that combining BRAF/MEK and immunotherapy is a feasible and active treatment option. In the context of an evolving treatment landscape with multiple immune checkpoint combinations now available, the future of the triplet regimen will depend on identifying the ideal clinical scenario for their use. A further biomarker‐driven investigation continues to identify patient subpopulations that could benefit from checkpoint inhibitor plus targeted therapy combinations.

## 5 CONCLUSIONS

In conclusion, in agreement with other reports, the present analysis indicated the efficacy of first‐line immunotherapy in patients with BRAF‐mutated malignant melanoma. We demonstrated that even anti‐PD‐1 monotherapy has good antitumor activity and might be considered for use in patients where doublet immunotherapy is not feasible.

The predictive value of the BCDRs could not be demonstrated. Based on these data, these BCDRs seem to have greater potential use for immunotherapy than targeted therapy. A further prospective trial is warranted to evaluate the potential predictive value of BDCR in the decision‐making process as alone standing parameter or in combination with other parameters such as PD‐L1 expression or gene signatures. Prospective trials should determine whether doublet immunotherapy or triplet therapy for patients with unfavourable clinical and laboratory parameters would improve patient outcomes.

## AUTHOR CONTRIBUTIONS

**Jindřich Kopecký:** Conceptualization (equal); data curation (equal); formal analysis (equal); methodology (equal); project administration (equal); supervision (equal); writing – original draft (equal); writing – review and editing (equal). **Marek Pásek:** Conceptualization (equal); data curation (equal); formal analysis (equal); investigation (equal); methodology (equal); writing – original draft (equal); writing – review and editing (equal). **Radek Lakomý:** Writing – review and editing (equal). **bohuslav melichar:** Writing – review and editing (equal). **Ivona Mrazová:** Data curation (equal); writing – review and editing (equal). **Ondřej Kubeček:** Conceptualization (equal); data curation (equal); methodology (equal); writing – review and editing (equal). **Monika Arenbergerová:** Resources (equal); writing – review and editing (equal). **Radmila Lemstrová:** Data curation (equal); resources (equal). **Alžběta Švancarová:** Data curation (equal); resources (equal). **Vojtěch Tretera:** Data curation (equal). **Alžběta Hlodáková:** Resources (equal). **Kamila Žváčková:** Resources (equal).

## FUNDING INFORMATION

This work was supported by the Cooperation Program, research area ONCO. This work was supported by the Charles University Faculty of Medicine in Hradec Kralove grant Progress Q40/06 and Q40/11.

## CONFLICT OF INTEREST STATEMENT

The authors declare no conflict of interest related to this article.

## ETHICS APPROVAL

The study was conducted following the Declaration of Helsinki and approved by the institutional ethic committee Ethics Committee University Hospital Hradec Králové.

## CONSENT TO PARTICIPATE

Patient consent was waived due to the REASON of retrospective design without changing the patient's therapy.

## CONSENT FOR PUBLICATION

Not applicable.

## Supporting information


Figure S1.

Figure S2.

Table S1.
Table S2.
Table S3.
Table S4.
Table S5.
Table S6.

## Data Availability

Research data are stored in an institutional repository and will be shared upon reasonable request to the corresponding author.
