# Supplementary material for: The outcome in patients with BRAF‐mutated metastatic melanoma treated with anti‐programmed death receptor‐1 monotherapy or targeted therapy in the real‐world setting
Source: Cancer Med. 2024 Mar 16;13(5):e6982. doi: 10.1002/cam4.6982 (PMC10943370; doi:10.1002/cam4.6982)
Supplement: Supplementary file 3 — Table S1. Table S2. Table S3. Table S4. Table S5. Table S6. [file CAM4-13-e6982-s001.docx]

|  | Immunotherapy | | Targeted therapy | | p-value |
| --- | --- | --- | --- | --- | --- |
|  | n= 47 | % | n= 90 | % | 0.002 |
| CR | 4 | 9 | 7 | 8 |  |
| PR | 10 | 21 | 42 | 47 |  |
| SD | 7 | 15 | 9 | 10 |  |
| Overall progression after DC | 21 | 45 | 58 | 64 |  |

Table S1. Progression rate after achieving disease control; CR – complete response, PR – partial response, SD – stable disease, DC – disease control.

|  | Immunotherapy | Targeted therapy |  |  |
| --- | --- | --- | --- | --- |
|  | Median (months, CI95%) | Median (months, CI95%) | HR (CI95%) | p-value |
| NLR ≥ cut-off | 7.0 (3.81-28) | 9.8 (7.00-14.00) | 1.15 (0.64-2.06) | 0.64 |
| NLR < cut-off | 25.8 (11.00-44.91) | 14.0 (10.00-19.00) | 0.66 (0.38-1.13) | 0.13 |
| LMR ≥ cut-off | 17.0 (8.00-44.91) | 13.0 (9.00-14.92) | 0.72 (0.47-1.12) | 0.14 |
| LMR < cut-off | 7.0 (1.51-39.00) | 12.0 (5.00-22.00) | 1.31 (0.52-3.26) | 0.57 |
| PLR ≥ cut-off | 8.0 (3.81-28.00) | 10.1 (7.20-15.08) | 1.02 (0.55-1.89) | 0.94 |
| PLR < cut-off | 20.9 (8.00-44.91) | 13.4 (9.79-16.00) | 0.69 (0.42-1.13) | 0.14 |
| SII ≥ cut-off | 7.0 (4.00-17.00) | 11.0 (7.00-14.00) | 1.16 (0.64-2.09) | 0.63 |
| SII < cut-off | 25.8 (11.96-44.91) | 14.0 (8.00-19.00) | 0.64 (0.38-1.08) | 0.09 |
| dNLR ≥ cut-off | 8.0 (4.00-25.80) | 11.0 (7.20-14.20) | 1.05 (0.62-1.78) | 0.85 |
| dNLR < cut-off | 44.9 (11.00-44.91) | 14.0 (8.00-19.00) | 0.60 (0.35-1.06) | 0.08 |
| LDH ≥ cut-off | 6.1 (3.06-28.00) | 9.0 (7.00-14.00) | 1.00 (0.58-1.75) | 0.98 |
| LDH < cut-off | 25.8 (8.00-44.91) | 13.4 (10.05-41.00) | 0.80 (0.46-1.40) | 0.43 |

Table S2. Kaplan-Mayer analysis for PFS comparing blood cell count-derived ratios; NLR – neutrophil-lymphocyte ratio, SII – systemic inflammation index, dNLR - derived neutrophil-lymphocyte ratio, LMR - lymphocyte-monocyte ratio, PLR - plate-let-lymphocyte ratio, LDH – lactate dehydrogenase, CI – confidence interval.

|  | | Immunotherapy + Targeted therapy | | | | | | |  |  |
| --- | --- | --- | --- | --- | --- | --- | --- | --- | --- | --- |
|  | CR | |  | PR |  | PD |  | CR+PR vs PD | | |
|  | n = 41 | | % | n = 70 | % | n = 37 | % | p-value | | |
| Sex |  | |  |  |  |  |  | 0.44 | | |
| Female | 17 | | 41 | 23 | 33 | 16 | 43 |  | | |
| Male | 24 | | 59 | 47 | 67 | 21 | 57 |  | | |
| Age |  | |  |  |  |  |  | 0.44 | | |
| ≥ 70 years | 15 | | 37 | 28 | 40 | 17 | 46 |  | | |
| < 70 years | 26 | | 63 | 42 | 60 | 20 | 54 |  | | |
| BRAF mutation |  | |  |  |  |  |  | 0.46 | | |
| V600E | 20 | | 49 | 39 | 56 | 20 | 54 |  | | |
| V600K | 7 | | 17 | 8 | 11 | 9 | 24 |  | | |
| Other (V600A, V600R G469K etc.) | 1 | | 2 | 1 | 1 | 0 | 0 |  | | |
| The type of mutation not assessed | 13 | | 32 | 22 | 31 | 8 | 22 |  | | |
| Synchronous metastatic disease (cut off 3 months) |  | |  |  |  |  |  | 0.12 | | |
| Yes | 9 | | 22 | 21 | 30 | 15 | 41 |  | | |
| No | 32 | | 78 | 49 | 70 | 22 | 59 |  | | |
| Number of involved organs |  | |  |  |  |  |  | 0.83 | | |
| 1 | 15 | | 37 | 14 | 20 | 8 | 22 |  | | |
| 2 and 3 | 22 | | 54 | 34 | 49 | 19 | 51 |  | | |
| ≥ 4 | 4 | | 10 | 22 | 31 | 10 | 27 |  | | |
| Metastatic site |  | |  |  |  |  |  | 0.09 | | |
| Lung | 10 | | 13 | 34 | 18 | 21 | 21 | 0.07 | | |
| Liver | 4 | | 5 | 21 | 11 | 12 | 12 | 0.23 | | |
| Lymphnodes | 25 | | 32 | 48 | 25 | 21 | 21 | 0.33 | | |
| Subcutaneous and soft tissue | 21 | | 27 | 36 | 19 | 19 | 19 | 1 | | |
| Brain | 2 | | 3 | 11 | 6 | 6 | 6 | 0.48 | | |
| Bone | 7 | | 9 | 14 | 7 | 9 | 9 | 0.48 | | |
| Other | 10 | | 13 | 25 | 13 | 10 | 10 | 0.61 | | |
| Adjuvant therapy |  | |  |  |  |  |  | 0.15 | | |
| Yes | 5 | | 12 | 6 | 9 | 7 | 19 |  | | |
| No | 36 | | 88 | 64 | 91 | 30 | 81 |  | | |
| LDH ≥ ULL |  | |  |  |  |  |  | 0.07 | | |
| Yes | 9 | | 22 | 38 | 54 | 22 | 59 |  | | |
| No | 32 | | 78 | 32 | 46 | 15 | 41 |  | | |
| NLR |  | |  |  |  |  |  | 0.88 | | |
| ≥ cut off | 13 | | 32 | 41 | 59 | 18 | 50 |  | | |
| < cut off | 28 | | 68 | 29 | 41 | 18 | 50 |  | | |
| LMR |  | |  |  |  |  |  | 0.76 | | |
| ≥ cut off | 34 | | 83 | 49 | 70 | 26 | 72 |  | | |
| < cut off | 7 | | 17 | 21 | 30 | 10 | 28 |  | | |
| PLR |  | |  |  |  |  |  | 0.65 | | |
| ≥ cut off | 13 | | 32 | 35 | 50 | 14 | 39 |  | | |
| < cut off | 28 | | 68 | 35 | 50 | 22 | 61 |  | | |
| SII |  | |  |  |  |  |  | 0.6 | | |
| ≥ cut off | 15 | | 37 | 40 | 57 | 16 | 44 |  | | |
| < cut off | 26 | | 63 | 30 | 43 | 20 | 56 |  | | |
| dNLR |  | |  |  |  |  |  | 0.81 | | |
| ≥ cut off | 25 | | 61 | 30 | 43 | 17 | 47 |  | | |
| < cut off | 16 | | 39 | 40 | 57 | 19 | 53 |  | | |

Table S3. Comparison of patients achieving complete and/or partial response with patients with progressive disease as the best objective response; CR – complete response, PR – partial response, PD – progressive disease, NLR – neutrophil-lymphocyte ratio, SII – systemic inflammation index, dNLR - derived neutrophil-lymphocyte ratio, LMR - lymphocyte-monocyte ratio, PLR - plate-let-lymphocyte ratio, ULL – upper limit level.

|  | Immunotherapy | | | | Targeted therapy | | | | p-value | | | |
| --- | --- | --- | --- | --- | --- | --- | --- | --- | --- | --- | --- | --- |
|  | CR  (n=17) | PR (n=16) | PD (n=18) | CR (n=24) | | PR (n=54) | PD (n=19) | CR  IO vs TT | | PR  IO vs TT | PD  IO vs TT |  |
|  | % | % | % | % | | % | % |  | |  |  |  |
| Sex |  |  |  |  | |  |  | 0.98 | | 0.65 | 0.89 |  |
| Female | 41 | 38 | 44 | 42 | | 31 | 42 |  | |  |  |  |
| Male | 59 | 63 | 56 | 58 | | 69 | 58 |  | |  |  |  |
| Histology |  |  |  |  | |  |  | 0.01 | | 0.54 | 0.23 |  |
| Nodular | 65 | 44 | 56 | 29 | | 41 | 37 |  | |  |  |  |
| Superficial spreading | 24 | 19 | 17 | 8 | | 9 | 26 |  | |  |  |  |
| Other | 0 | 19 | 22 | 8 | | 15 | 11 |  | |  |  |  |
| Metastatic | 12 | 19 | 6 | 54 | | 35 | 26 |  | |  |  |  |
| Age |  |  |  |  | |  |  | 0.61 | | 0.82 | 0.14 |  |
| ≥ 70 years | 41 | 38 | 33 | 33 | | 41 | 58 |  | |  |  |  |
| < 70 years | 59 | 63 | 67 | 67 | | 59 | 42 |  | |  |  |  |
| Localisation of the primary tumour |  |  |  |  | |  |  | 0.45 | | 0.31 | 0.37 |  |
| Limbs | 35 | 44 | 33 | 29 | | 24 | 32 |  | |  |  |  |
| Trunk | 35 | 44 | 44 | 50 | | 50 | 32 |  | |  |  |  |
| Head | 18 | 0 | 17 | 4 | | 11 | 11 |  | |  |  |  |
| Metastatic | 12 | 13 | 6 | 17 | | 15 | 26 |  | |  |  |  |
| BRAF mutation |  |  |  |  | |  |  | 0.004 | | 0.14 | 0.32 |  |
| V600E | 53 | 69 | 61 | 46 | | 52 | 47 |  | |  |  |  |
| V600K | 35 | 6 | 28 | 4 | | 13 | 21 |  | |  |  |  |
| Other (V600A, V600R G469K etc.) | 6 | 6 | 0 | 0 | | 0 | 0 |  | |  |  |  |
| The type of mutation not assessed | 6 | 19 | 11 | 50 | | 35 | 32 |  | |  |  |  |
| Synchronous metastatic disease (cut off 3 months) |  |  |  |  | |  |  | 0.84 | | 0.46 | 0.84 |  |
| Yes | 24 | 38 | 39 | 21 | | 28 | 42 |  | |  |  |  |
| No | 76 | 63 | 61 | 79 | | 72 | 58 |  | |  |  |  |
| Number of involved organs |  |  |  |  | |  |  | 0.21 | | 0.06 | 0.24 |  |
| 1 | 41 | 38 | 33 | 33 | | 15 | 11 |  | |  |  |  |
| 2 and 3 | 59 | 50 | 44 | 50 | | 48 | 58 |  | |  |  |  |
| ≥ 4 | 0 | 13 | 22 | 17 | | 37 | 32 |  | |  |  |  |
| Metastatic site |  |  |  |  | |  |  | 0.19 | | 0.15 | 0.25 |  |
| Lung | 11 | 14 | 23 | 14 | | 19 | 20 | 0.4 | | 0.12 | 0.89 |  |
| Liver | 4 | 5 | 9 | 6 | | 13 | 15 | 0.49 | | 0.08 | 0.2 |  |
| Lymphnodes | 39 | 30 | 14 | 27 | | 24 | 27 | 0.68 | | 0.99 | 0.006 |  |
| Subcutaneous and soft tissue | 29 | 24 | 28 | 25 | | 18 | 13 | 0.66 | | 0.66 | 0.07 |  |
| Brain | 4 | 3 | 5 | 2 | | 7 | 7 | 0.8 | | 0.24 | 0.42 |  |
| Bone | 4 | 11 | 12 | 12 | | 7 | 7 | 0.11 | | 0.57 | 0.64 |  |
| Other | 11 | 14 | 9 | 14 | | 13 | 11 | 0.4 | | 0.67 | 0.53 |  |
| Adjuvant therapy |  |  |  |  | |  |  | 0.3 | | 0.71 | 0.62 |  |
| Yes | 6 | 6 | 22 | 17 | | 9 | 16 |  | |  |  |  |
| No | 94 | 94 | 78 | 83 | | 91 | 84 |  | |  |  |  |
| LDH ≥ ULL |  |  |  |  | |  |  | 0.83 | | 0.04 | 0.64 |  |
| Yes | 24 | 31 | 56 | 21 | | 61 | 63 |  | |  |  |  |
| No | 76 | 69 | 44 | 79 | | 39 | 37 |  | |  |  |  |
| NLR |  |  |  |  | |  |  | 0.02 | | 0.17 | 0.74 |  |
| ≥ cut off | 12 | 44 | 53 | 46 | | 63 | 47 |  | |  |  |  |
| < cut off | 88 | 56 | 47 | 54 | | 37 | 53 |  | |  |  |  |
| SII |  |  |  |  | |  |  | 0.04 | | 0.22 | 0.34 |  |
| ≥ cut off | 18 | 44 | 53 | 50 | | 61 | 37 |  | |  |  |  |
| < cut off | 82 | 56 | 47 | 50 | | 39 | 63 |  | |  |  |  |
| dNLR |  |  |  |  | |  |  | 0.29 | | 0.51 | 0.04 |  |
| ≥ cut off | 71 | 50 | 29 | 54 | | 41 | 63 |  | |  |  |  |
| < cut off | 29 | 50 | 71 | 46 | | 59 | 37 |  | |  |  |  |
| LMR |  |  |  |  | |  |  | 0.45 | | 0.02 | 0.59 |  |
| ≥ cut off | 88 | 94 | 76 | 79 | | 63 | 68 |  | |  |  |  |
| < cut off | 12 | 6 | 24 | 21 | | 37 | 32 |  | |  |  |  |
| PLR |  |  |  |  | |  |  | 0.35 | | 0.26 | 0.35 |  |
| ≥ cut off | 24 | 38 | 47 | 38 | | 54 | 32 |  | |  |  |  |
| < cut off | 76 | 63 | 53 | 63 | | 46 | 68 |  | |  |  |  |

Table S4. Comparison of patients achieving complete, partial and progressive response between groups treated with immunotherapy and targeted therapy; CR – complete response, PR – partial response, PD – progressive disease, NLR – neutrophil-lymphocyte ratio, SII – systemic inflammation index, dNLR - derived neutrophil-lymphocyte ratio, LMR - lymphocyte-monocyte ratio, PLR - plate-let-lymphocyte ratio, ULL – upper limit level.

|  | Univariant analysis | | | Multivariant analysis | | |
| --- | --- | --- | --- | --- | --- | --- |
|  | HR | 95%CI | P value | HR | 95%CI | p-value |
| Gender (women vs men) | 0.92 | 0.57-1.50 | 0.74 | --- | --- | --- |
| Age ≥ 70 (yes vs no) | 0.92 | 0.56-1.5 | 0.74 | --- | --- | --- |
| Initial metastatic (yes vs no) | 1.01 | 0.61-1.69 | 0.96 | --- | --- | --- |
| Number of involved sites ≥ 3 (yes vs no) | 1.61 | 1.01-2.59 | 0.049 | 1.09 | 0.64-1.87 | 0.75 |
| Adjuvant therapy (yes vs no) | 1.16 | 0.64-2.11 | 0.63 | --- | --- | --- |
| LDH ≥ ULL (no vs yes) | 0.58 | 0.36-0.91 | 0.02 | 0.57 | 0.36-0.92 | 0.02 |
| NLR1 ≥ 3 (no vs yes) | 0.81 | 0.52-1.28 | 0.37 | --- | --- | --- |
| LMR1 ≥ 2 (no vs yes) | 1.05 | 0.65-1.71 | 0.84 | --- | --- | --- |
| PLR1 ≥ 160 (no vs yes) | 0.92 | 0.59-1.44 | 0.71 | --- | --- | --- |
| SII1 ≥ 800 (no vs yes) | 0.86 | 0.55-1.35 | 0.51 | --- | --- | --- |
| dNLR1 ≥ 1,9 (no vs yes) | 0.83 | 0.53-1.31 | 0.43 | --- | --- | --- |
| Lung metastases (yes vs no) | 1.92 | 1.23-3.03 | 0.004 | 1.73 | 1.06-2.83 | 0.03 |
| Liver metastases (yes vs no) | 1.66 | 1.03-2.69 | 0.038 | 1.13 | 0.66-1.94 | 0.66 |
| Lymphnodes metastases (yes vs no) | 1.35 | 0.83- 2.21 | 0.23 | --- | --- | --- |
| Subcutaneous metastases (yes vs no) | 1.09 | 0.70-1.71 | 0.69 | --- | --- | --- |
| Brain metastases (yes vs no) | 2.01 | 1.09-3.70 | 0.02 | 1.94 | 1.04-3.61 | 0.04 |
| Bone metastases (yes vs no) | 1.03 | 0.58-1.82 | 0.92 | --- | --- | --- |

Table S5. Univariate and multivariate analysis for progression-free survival in targeted therapy group only; NLR – neutrophil-lymphocyte ratio, SII – systemic inflammation index, dNLR - derived neutrophil-lymphocyte ratio, LMR - lymphocyte-monocyte ratio, PLR - platelet-lymphocyte ratio, IO – immunotherapy, BRAF – BRAF inhibition, CI – confidential interval.

|  | Univariant analysis | | | Multivariant analysis | | |
| --- | --- | --- | --- | --- | --- | --- |
|  | HR | 95%CI | P value | HR | 95%CI | p-value |
| Gender (women vs men) | 1.49 | 0.79-2.81 | 0.21 | --- | --- | --- |
| Age ≥ 70 (yes vs no) | 0.37 | 0.17-0.80 | 0.01 | 0.4 | 0.18-0.90 | 0.02 |
| Initial metastatic (yes vs no) | 1.35 | 0.70-2.61 | 0.37 | --- | --- | --- |
| Number of involved sites ≥ 3 (yes vs no) | 2.03 | 0.84-4.86 | 0.11 | --- | --- | --- |
| Adjuvant therapy (yes vs no) | 2 | 0.78-5.19 | 0.15 | --- | --- | --- |
| LDH ≥ ULL (yes vs no) | 2.08 | 1.10-3.93 | 0.02 | 2.24 | 1.11-4.53 | 0.02 |
| NLR1 ≥ 3 (yes vs no) | 1.89 | 1.00-3.59 | 0.04 | 1.12 | 0.52-2.37 | 0.77 |
| LMR1 ≥ 2 (yes vs no) | 0.56 | 0.24-1.26 | 0.16 | --- | --- | --- |
| PLR1 ≥ 160 (yes vs no) | 1.47 | 0.77-2.82 | 0.24 | --- | --- | --- |
| SII1 ≥ 800 (yes vs no) | 1.83 | 0.97-3.46 | 0.06 | --- | --- | --- |
| dNLR1 ≥ 1,9 (yes vs no) | 1.9 | 1.01-3.59 | 0.046 | 0.87 | 0.31-2.45 | 0.79 |
| Lung metastases (yes vs no) | 1.43 | 0.75-2.72 | 0.27 | --- | --- | --- |
| Liver metastases (yes vs no) | 1.69 | 0.74-3.84 | 0.21 | --- | --- | --- |
| Lymphnodes metastases (yes vs no) | 0.53 | 0.28-1.0 | 0.048 | 1.84 | 0.90-3.76 | 0.09 |
| Subcutaneous metastases (yes vs no) | 1.94 | 1.03-3.69 | 0.04 | 2.35 | 1.19-4.63 | 0.01 |
| Brain metastases (yes vs no) | 0.49 | 0.12-2.04 | 0.33 | --- | --- | --- |
| Bone metastases (yes vs no) | 2.05 | 0.97-4.33 | 0.06 | --- | --- | --- |

Table S6. Univariate and multivariate analysis for progression-free survival in the immunotherapy group only; NLR – neutrophil-lymphocyte ratio, SII – systemic inflammation index, dNLR - derived neutrophil-lymphocyte ratio, LMR - lymphocyte-monocyte ratio, PLR - platelet-lymphocyte ratio, IO – immunotherapy, BRAF – BRAF inhibition, CI – confidential interval.
